# Increased Plasma Osteocalcin, Oral Disease, and Altered Mandibular Bone Density in Postmenopausal Women

**DOI:** 10.1155/2019/3715127

**Published:** 2019-10-24

**Authors:** Supanee Thanakun, Suchaya Pornprasertsuk-Damrongsri, Chantida Pawaputanon Na Mahasarakham, Suteera Techatanawat, Yuichi Izumi

**Affiliations:** ^1^College of Dental Medicine, Rangsit University, Pathum Thani 12000, Thailand; ^2^Oral Diagnosis and Oral Medicine Clinic, Dental Hospital, Faculty of Dentistry, Mahidol University, Bangkok 10400, Thailand; ^3^Department of Oral and Maxillofacial Radiology, Faculty of Dentistry, Mahidol University, Bangkok 10400, Thailand; ^4^Department of Restorative Dentistry, Faculty of Dentistry, Khonkaen University, Khonkaen 40000, Thailand; ^5^Department of General Dentistry, Faculty of Dentistry, Srinakharinwirot University, Bangkok 10110, Thailand; ^6^Department of Periodontology, Graduate School of Medical and Dental Sciences, Tokyo Medical and Dental University, Tokyo, Japan; ^7^Oral Care Perio Center, Southern Tohoku General Hospital, Southern Tohoku Research Institute for Neuroscience, Fukushima, Japan

## Abstract

An association between oral diseases and postmenopausal status has been recognized. However, the relationship between all oral disease, mandibular bone density, health status, and osteocalcin (OCN) bone markers in postmenopausal dental patients has not been reported. This study was therefore to verify the differences in plasma OCN levels, dental, periodontal, and oral mucosal disease, and mandibular bone density alterations from panoramic radiograph and systemic parameters in postmenopausal women, compared to premenopausal women. Oral, radiographic, and blood examination were performed in 92 females. Dental, periodontal, and oral mucosal statuses were recorded. Health profile parameters were collected from medical charts. Plasma OCN was evaluated by enzyme-linked immunosorbent assay. Forty-two (45.7%) participants were postmenopausal with a higher median age (55 (51, 62) years) than the premenopausal group (43 (38, 45) years). Overweight or obesity, hypercholesterolemia, and impaired fasting blood sugar were more prevalent in postmenopause. The average postmenopausal OCN level (425.62 ng/mL) was significantly higher than the premenopausal group (234.77 ng/mL, *p* < 0.001). The average number of missing teeth, mean attachment loss, alveolar bone loss, periapical lesion count, and clinical oral dryness score were also significantly higher in postmenopause (*p*=0.008,   < 0.001,  0.031,  0.006,  and 0.005, respectively). However, mandibular bone density determined by mandibular cortical index was lower in postmenopause (*p* < 0.001). The panoramic mandibular index, mandibular cortical width, fractal dimension, and other oral mucosal disease did not differ between the groups. Postmenopause was associated with elevated plasma OCN (*β* = 0.504, *p* < 0.001) when related covariates were adjusted. Elevated plasma OCN, oral mucosal dryness, high number of periapical radiolucencies and missing teeth, and lower mandibular bone density from panoramic radiograph were prevalent in postmenopausal women. Dentists should suspect an increased risk of low bone mineral density in postmenopausal patients who display these clinical and radiographic findings, and they should be referred for further examination. Plasma OCN may interconnect a relationship between postmenopausal status and the low mandibular bone density.

## 1. Introduction

Many biological changes occur in postmenopausal women, and the majority of these changes are because of decreased estrogen production. Oral mucosa, osteoblasts, and fibroblasts in periodontal tissue contain estrogen receptors [1, 2]. Estrogen deficiency can therefore cause a range of disorders, including osteopenia and osteoporosis as well as oral changes. The role of estrogen in maintaining oral mucosal, dental, and periodontal health in postmenopausal women is not clear. Particularly, only few number of studies regarding the effects of menopause on the oral mucosa are available [3, 4]. Many studies show that osteoporosis in postmenopausal women also affects oral bone and is a perpetuating factor for periapical and periodontal disease, including tooth loss, if etiological factors are present [5–7]. Additionally, past researchers reported the link between decreased mandibular bone mineral density (BMD) and menopause [8]. Thus, oral health can be disturbed in postmenopausal women, and this requires attention in addition to the other important issues associated with menopause.

Dual-energy X-ray absorptiometry (DXA) is considered the gold standard for BMD assessment in the vertebrae, femoral neck, and forearms [9]; however, research into several panoramic radiography indices has been performed to identify a predictor of low BMD, so that the dentist can play an important role in screening patients with low BMD and referring them appropriately for osteopenia and osteoporosis investigation. BMD in the mandible has been shown to be positively correlated with that in the lumbar spine, femoral neck, and forearm, which are important sites for osteoporosis [8, 10, 11]. However, as none of the indices investigated have perfect sensitivity and specificity in detecting osteopenia or osteoporosis in the mandible, combining them with clinical indices has been proposed [8]. It would be useful to further study the possibility of relating the different indices with clinical parameters to detect osteopenia and osteoporosis in the mandible in postmenopausal dental patients.

Osteocalcin (OCN), produced exclusively by osteoblasts, is involved in bone formation and calcium homeostasis [12]. OCN also plays a crucial role as a hormone that impacts glucose metabolism, energy homeostasis, reproduction, and recognition [12]. Circulating OCN levels are associated with abdominal obesity, metabolic syndrome, type 2 diabetes, and decreased BMD [12–14]. OCN is synthesized during bone formation, and it exhibits a compact, calcium-dependent, alpha-helical conformation, in which the gamma carboxyglutamic acid residues bind and promote absorption to hydroxyapatite in the bone matrix. In this way, bone mineralization takes place. Nevertheless, in most bone remodeling circumstances, bone formation remains at least partially coupled to bone resorption. OCN is released from the bone matrix into the blood during bone resorption. It is suggested that OCN is a marker of bone turnover rather than bone formation [15]. It is assumed that while the bone turnover rate is constant in premenopausal women, it is stimulated during the postmenopausal period, resulting in elevated OCN levels. In osteoporotic women, the reduced formation of hydroxyapatite crystals stimulates free OCN to circulate in the blood. This may explain the increased concentrations of OCN in the plasma of osteoporotic postmenopausal women [16]. Quantifying plasma OCN concentrations may, therefore, be helpful to monitor early changes that cannot be detected with BMD assessment. The elevation of OCN levels could be a more efficient method for early detection in patients with rapid bone turnover rates after the onset of menopause [17].

In contrast to the various documents available on BMD assessment from dental radiographs, no data exist on the relationship between oral diseases, panoramic radiograph (PAN) assessment for screening of BMD, health status, and bone markers, especially OCN, in postmenopausal dental patients. Therefore, the objective of the current study was to examine the possible association of postmenopause with oral and systemic health parameters. We also investigated whether there is an underlying relationship between plasma OCN and mandibular bone alteration in postmenopausal women with the definitive aim of providing broader knowledge about postmenopausal dental patients.

## 2. Materials and Methods

### 2.1. Study Population

Ninety-two females who were examined in the Golden Jubilee Medical Center, Mahidol University, and provided a history of postmenopausal status (menopause had occurred at least 1 year before their visit) were included. This study investigated the same group as our previously published study [18]. Pre- or postmenopausal females aged 35–75 years consented to participate in this study. All of them did not use hormone replacement therapy. Except one participant (2.4%) had been receiving estrogen replacement therapy for less than 1 year. All participants had no history of systemic disease, medication use, radiation, or chemotherapy. Exclusion criteria comprised women who had received medication or had a history and/or the presence of other infections, or who had received systemic antibiotics, immunosuppressive drugs, or periodontal treatment in the 6 months prior to recording.

This study was approved by the Ethics Committee of Mahidol University and Tokyo Medical and Dental University and conformed with the Declaration of Helsinki (reference number: MU-IRB 2011/134.3006 and TMDU-IRB 2012/860).

### 2.2. Clinical Assessments

Data collected from the patients' medical charts included age, body mass index (BMI) plus waist circumference, cholesterol, high-density lipoprotein cholesterol, low-density lipoprotein cholesterol, triglyceride, fasting blood sugar, systolic/diastolic blood pressure (BP), and glomerular filtration rate. BMI ≥23 kg/m^2^ plus waist circumference ≥85 cm in males or ≥80 cm in females was diagnosed as overweight or obesity. The blood tests were analyzed using routine methods. A standard mercury sphygmomanometer was used for BP measurements. BP was measured two consecutive times after the participants rested for at least 5 min, and the average value was used for the analysis. Information on personal habits (alcohol consumption and smoking: never, former, and current) was collected from interview.

### 2.3. Oral Examination

Oral examination consisted of diagnoses of oral mucosal health and dryness. The dental and periodontal status was also assessed by an oral diagnosis and oral medicine specialist (ST).

Clinical score of oral dryness (CSOD) was evaluated in each participant to assess oral mucosal dryness. CSOD was based on 10 key features of dry mouth with sample images, and one point was allocated for each feature to assess the presence of dry mouth. A low CSOD score (1–3) indicates mild dryness that is normally manageable in practice, whereas a high CSOD score (7–10) indicates that further investigation is required [19].

The number of missing teeth, excluding the third molars, was examined in a standard dental unit during the same visit as the radiographic examination. For recording periodontal status, full-mouth periodontal examination, excluding the third molars, was performed with a standardized method using a manual 12 UNC colour-coded periodontal probe (Hu-Friedy, Chicago, IL, USA). Details on the examination of periodontal disease have been previously reported [20].

### 2.4. Periapical Radiolucency, Alveolar Bone Loss, and Mandibular BMD Evaluation

Digital PANs were obtained for all participants at the same visit as the oral examination using an extraoral panoramic X-ray unit (Planmeca Proline XC, Helsinki, Finland) with the following exposure settings: 8 mA, 66–70 kV, and 18 sec. The participants were positioned in the panoramic unit so that the vertical line produced by the unit was aligned with the facial midline and the horizontal line (Frankfort plane) was parallel to the floor. Head alignment, film density, and participant positioning were within the reviewer's standard range of quality. The periapical lesions, alveolar bone loss (ABL), and mandibular BMD indices were assessed on PAN by an oral-maxillofacial radiologist (SPD) with more than 10 years of experience in radiology.

The number of diseased teeth with periapical radiolucencies was diagnosed on the basis of PAN examination along with information from history taking and intraoral clinical examination. Radiolucent periapical lesions were assessed after careful analysis of the periodontal ligament space, lamina dura, trabecular pattern, and bone marrow spaces, according to the criteria previously described by Halse and Molven [21].

ABL was radiographically measured on the mesial and distal surfaces of six teeth using Image J (version 1.49a; National Institutes of Health, Bethesda, MD, USA), and the ABL percentage was calculated according to Beckstrom et al. [22]. The full method was described in our previous study [18].

**Figure 1:**
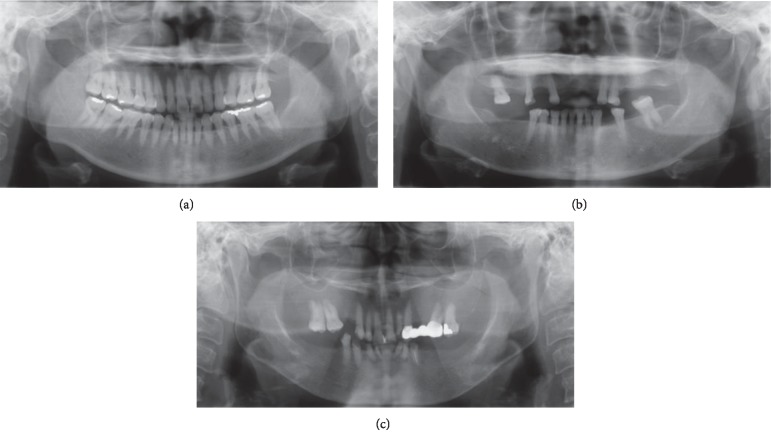
Mandibular cortical index (MCI): C1, the endosteal margin of the cortex is even and sharp on both sides (a); C2, the endosteal margin forms one layer of endosteal cortical residues on both sides (b); and C3, the cortical layer forms heavy endosteal residues and is clearly thin on both sides (c).

The following indices for mandibular BMD evaluation were measured on each PAN:

1. Mandibular cortical index (MCI): according to the classification of Klemetti et al. [23], MCI is the appearance of the inferior mandibular cortical thickness, which is as follows: C1: the endosteal margin of the cortex is even and sharp on both sides, C2: the endosteal margin shows semilunar defects (lacunar resorption) or seems to form endosteal cortical residues (one to three layers) on one or both sides, and C3: the cortical layer forms heavy endosteal residues and is clearly porous (Figure 1).
2. Panoramic mandibular index (PMI): the PMI is the ratio of the thickness of the mandibular cortex to the distance between the inferior margin of the mental foramen and the inferior mandibular cortex [24].
3. Mandibular cortical width (MCW): mandibular cortical thickness was measured on the line that was perpendicular to the inferior border of the mandible at the middle of the mental foramen [25].
4. Fractal dimension (FD): the FD analysis was modified from Koh et al. [26] and from Yaşar and Akgünlü [27]. First, the 8-bit direct digital radiograph was exported from the server and opened with Image J (version 1.5i). Square regions of interest (ROIs) with the same dimensions of 51 × 51 pixels were created between the apical roots of the first and second premolars and considered as the original image. Second, each ROI was blurred through the use of a Gaussian filter with a radius of 35 pixels. The resulting blurred image was then subtracted from the original image, and a gray value of 128 was obtained. Third, the generated image was then made binary, eroded, dilated, and skeletonized. Finally, the skeletonized image was calculated for the fractal dimension value using the box counting method in the Image J program.

### 2.5. Blood Collection and OCN Analyses

Peripheral venous blood samples from each participant were collected between 9:00 A.M. and 12:00 P.M. after overnight fasting. To avoid repetitive freeze-thaw cycles, many aliquots of one sample, from the same group of patients as in our previously published study, were arranged and stored at −80°C until analyses [18]. OCN levels were measured with an enzyme-linked immunosorbent assay kit (SimpleStep ELISA®; Abcam®, Cambridge, UK), following the manufacturer's instructions. This kit employs an affinity tag-labeled capture antibody and a reporter-conjugated detector antibody that immunocapture the sample analyte in solution. This entire complex (capture antibody/analyte/detector antibody) is in turn immobilized via the immunoaffinity of an anti-tag antibody coating the well. Briefly, samples of 50 *μ*L at 100-fold dilution or 0–10 ng/mL standards were added to the wells, followed by 50 *μ*L of the antibody cocktail, and were incubated for 1 h at room temperature on a plate shaker set to 400 rpm. After three washes, 100 *μ*L of the tetramethylbenzidine substrate was added to each well and incubated for 10 min in a dark room on a plate shaker set to 400 rpm. Finally, 100 *μ*L of stop solution was added to each well, the microplate was placed on a plate shaker for 1 min to mix, and the optical density was recorded at 450 nm by a microplate reader (SOFTMax™ Molecular Devices Corp., CA, USA). A four parameter logistic provided the curve fit, and the amount of OCN present in the plasma samples was calculated.

### 2.6. Statistical Analyses

All statistical analyses were performed using IBM SPSS Statistics for Windows, Version 22.0 (IBM Corp, Armonk, NY, USA). The normality of all continuous variables was assessed using the Kolmogorov–Smirnov test. The comparison of characteristics between the categories of pre- or postmenopausal status was performed using the chi-squared or Mann–Whitney *U* test for qualitative or quantitative data, respectively, since the normality assumption was not satisfied. Data were first summarized by median and interquartile range (if continuous) or frequency and percentage (if categorical). The Spearman correlation coefficients were calculated to determine correlation among all parameters. Thereafter, stepwise multiple linear regression analyses were conducted to test the relationship between the effect of potential prognostic factors on the presence of elevated OCN levels and statistically significant factors from bivariate analysis forced into the model, and this was expressed as *β*. *p* value <0.05 was considered statistically significant.

## 3. Results

The distribution of health-related characteristics and personal history of the participants is shown in Table 1. Among the 92 participants involved in the study, 42 (45.7%) were postmenopausal and 50 (54.3%) were premenopausal. The median (1^st^ and 3^rd^ quartile) age of the postmenopausal women (55 (51, 62) years) was higher than the premenopausal women (43 (38, 45) years). The number of women with alcohol consumption and current smoking between the groups did not differ significantly. Overweight or obesity, hypercholesterolemia, and impaired fasting blood sugar conditions were more prevalent in the postmenopausal group. The average OCN level in the postmenopausal group was 425.62 ng/mL, as compared with 234.77 ng/mL in the premenopausal group.

The average number of missing teeth between the two studied groups was 6 (2, 16) and 1 (2, 4), with significantly more missing teeth in the postmenopausal group (*p*=0.008). The mean attachment loss (AL), ABL, and number of periapical lesions were analyzed, and all of these oral disease parameters showed similar differences, as they were more predominant in the postmenopausal women (Table 2). CSOD was also significantly higher in the postmenopausal group (Table 2). However, other oral mucosal diseases of the two studied groups were not different.

**Figure 2:**
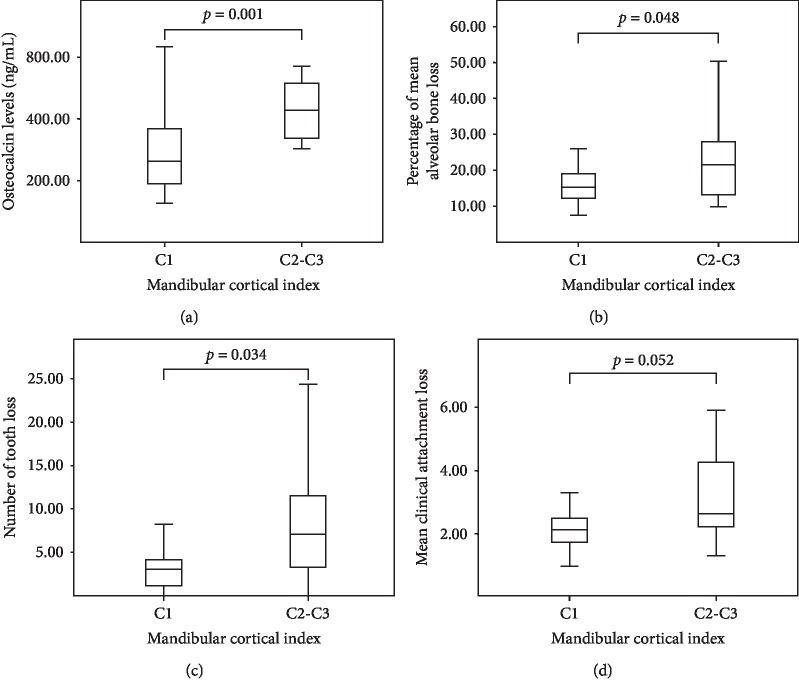
Levels of plasma OCN (a), mean ABL (b), number of missing teeth (c), and mean AL (d) according to the MCI. The results of Mann–Whitney *U* test showed significant differences, as women with MCI C2 and C3 had higher OCN levels (*p* < 0.001), ABL (*p*=0.048), and tooth loss (0.034) than those with MCI C1. AL did not differ significantly among the MCI groups (0.052).

Regarding the decreased mandibular BMD from PANs, the MCIs on both the left and right sides were higher in postmenopausal women (*p* < 0.001). However, there were no significant differences in PMI, MCW, or FD between the groups (Table 3). When MCI was considered, women with MCI C2 and C3 had higher OCN levels (*p* < 0.001), bone loss, tooth loss, and AL (*p*=0.048, 0.034,  and 0.052, respectively) than those with MCI C1 (Figure 2).

Finally, the association of elevated plasma OCN levels with the participants' general and oral health status was analyzed by multivariate analysis. Because of correlation between tooth loss and bone loss (*p*=0.006), only bone loss was an independent variable entered into the model. Postmenopausal status was associated with elevated plasma OCN levels (*β* = 0.504, *p*=0.001) when age, ABL, MCI, and health status of the participants were adjusted (Table 4).

## 4. Discussion

The results of this study showed that postmenopausal patients had a higher tendency for oral mucosal dryness, disease of the periapical tissue, AL, ABL, and tooth loss than premenopausal women. We observed decreased mandibular BMD evaluated by MCI in this postmenopausal group. Dyslipidemia, impaired fasting sugar, and overweight or obesity status were also noted. Data additionally exhibited a positive association between elevated levels of plasma OCN and postmenopausal status after influencing factors adjusted. This study is, therefore, a report of all dental, periodontal, and oral mucosal disease, including the health profile and bone turnover marker, OCN, in postmenopausal versus premenopausal dental patients.

Because the oral mucosa and salivary glands contain estrogen receptors, changes in the level of estrogen in postmenopausal women may affect the oral cavity [1]. Minicucci et al. reported reduction in salivary flow rate without clinical symptoms of dry mouth in postmenopausal women [4]. Agha-Hosseini et al. studied the relationship between lumbar spine BMD and oral dryness in 60 menopausal women [3]. They observed significant negative correlation between lumbar spine BMD and oral dryness score. The BMD was significantly lower in postmenopausal women with xerostomia and reduced salivary flow rate in unstimulated condition [3]. The CSOD used in the present study is accurately related to both the unstimulated salivary flow and the severity of dry mouth [19]. In the current study, although the other oral mucosal disease was not different from that of premenopausal women, it can be proposed that postmenopausal women with higher oral dryness experience had reduced salivary flow rate as determined by CSOD. Therefore, it is important to normalize salivary flow to prevent oral disorders and maintain oral health by eliminating possible causes for the reduction of salivary flow rates, advising patients for frequent water sipping and/or artificial saliva use. The administration of hormone replacement therapy (HRT) to postmenopausal women was reported to be useful for preventing osteoporosis and indirectly increasing the salivary flow rate [28]. However, data on the effect of menopause on saliva are based on small patient numbers, and there are no randomized controlled trials on the effect of HRT on salivary secretion and composition. Hence, well-designed and adequately powered studies are required.

In the present study, one third of postmenopausal women showed 1–4 teeth with periapical radiolucencies, whereas only one tooth with periapical radiolucency was found in 16% of the premenopausal group. Our significant result is comparable to the study by López-López et al. [5], who found that low BMD is marginally associated with a higher frequency of radiolucent periapical lesions [5]. Brasil et al. researched ovariectomized rats and reported that estrogen deficiency resulted in significantly greater body mass gain and significantly larger apical periodontitis lesions when compared with controls [29]. We found very scarce articles addressing this issue; thus, more studies are needed to further clarify any association. However, alterations in bone metabolism that are typical of postmenopausal women contribute to bone loss and reduced bone repair ability. These hypotheses may help explain the findings of the present study.

The results of the latest meta-analysis showed that women with osteopenia and osteoporosis present with greater mean AL in comparison with women with normal BMD [30]. This publication has indicated that low BMD has an effect on AL, which is similar to the trend found in our study. A positive correlation between systemic osteoporosis and ABL has been reported [7, 31]. Decreased alveolar BMD can lead to tooth loss. A significant inverse correlation was found between BMD in the hip region and the number of missing teeth [6]. Makker et al. reported an increase in the risk of tooth loss with decreased mandibular BMD [11]. Lee et al. showed a positive association between osteoporosis and periodontitis (OR = 1.21) in the general population after adjustment for age [32]. Similarly, a possible association between osteoporosis in postmenopausal women and periodontitis has been reported [6, 7]. Postmenopausal women present with periodontal disease more frequently and in a more severe form than premenopausal women [7]. ABL and tooth loss is a principal sign of periodontitis in Thai people [33]. Similarly to the present study, in the study by Singh et al., the AL and ABL of postmenopausal women were found to be negatively and significantly correlated with skeletal BMD [7]. A greater predilection to lose alveolar bone in postmenopausal women with osteoporosis, especially in women with preexisting periodontitis, is postulated. It was hypothesized that low systemic BMD may directly affect the microarchitecture of alveolar bone, possibly influencing the rate of periodontal destruction in periodontitis and leading to bone loss and tooth loss [2]. Longitudinal studies are lacking and additionally needed to prove a causal relationship between osteoporosis and periodontitis.

As stated earlier, osteoporosis is a major health problem in postmenopausal women. It is diagnosed on clinical suspicion and BMD measurement. DXA scan is the gold standard for diagnosis [9]. Few studies have reported BMD measurement performed in the body of the mandible by DXA [11, 34, 35]. The standard mandibular site (with the smallest possible inter- and intraindividual variations in anatomical size, shape, bone structure, and function) is the basal area of the mandible posterior to the mental foramen. It is concluded that mandibular BMD assessed by DXA correlates significantly with BMD measurements of other important skeletal sites [11, 34]. Therefore, in places where DXA facilities are either not available or are beyond the affordable limit, PAN is still practicable. Gulsahi et al. disclosed conflicting results that the BMD of the maxilla or mandible was not correlated with either MCI and PMI or femoral BMD [35].

MCI can be reliably used as a diagnostic tool for screening postmenopausal women with osteoporosis [36]. The presence of any kind of cortical erosion (C2 or C3) can be considered a useful indicator of reduced BMD because in nearly 80% of cases it is associated with at least osteopenia [8]. Gaur et al. demonstrated a significant relationship between BMD and MCI. In their study, the specificity was 88.9% and the sensitivity was 100% [37]. Studies investigating the accuracy of the MCI in detecting reduced BMD had rather homogeneous results [38]. This was in accordance with our findings that MCI differed significantly between postmenopausal and premenopausal women [36]. Postmenopausal women had a thinner or more eroded cortex of the mandible detected on PAN than premenopausal women. Furthermore, tooth loss and bone loss were significantly higher in postmenopausal women with MCI C2 and C3 in the present study.

Although correlation of PMI and BMD are controversial [38], from the systematic review and meta-analysis of Calciolari et al., PMI with a cutoff value of 0.3 seems to be the most accurate linear index to screen for reduced BMD [8]. The mean values of PMI in both groups of the current study were 0.3; therefore, assessment of differently reduced BMD was consequently impracticable. Less strong conclusions can be drawn for MCW [8]. The MCW of the osteopenic/osteoporotic groups was lower than that of the healthy group. Taguchi et al. reported that MCW below 3 mm may be considered a threshold value when predicting osteoporosis or osteopenia and is a criterion for referring patients for BMD evaluation [39]. MCW presented with a better accuracy in excluding osteopenia and osteoporosis and is more useful to exclude high risk for low BMD because in 90% of cases, patients with a cortical width wider than 4 mm have a normal BMD [8]. These cut points might explain why MCW showed insignificant results in the present study. The average values for MCW in both studied groups were less than 4 mm. Nonetheless, MCW was likely to be lower in the postmenopausal versus the premenopausal group in the current study.

Studies have reported differences in FD between healthy and osteoporotic patients; however, a previous review presented controversial results for the correlation of mandibular FD with skeletal BMD [38]. There was also a significant difference in the FD values among different jaw sites. Our study similarly showed unbalanced results between right and left mandibular BMD; the difference between the two studied groups was consequently unable to be determined. Further investigation should therefore be continued in a larger population to verify this index for screening low BMD from PAN.

In the study by Kavitha et al., the combination of MCW from digital PAN with FD and the gray level co-occurrence matrix method demonstrated the usefulness of these mandibular bone textural feature evaluations for discriminating individuals with low BMD from healthy persons by comparing particular textural features or MCW [40]. The use of recent technology, such as microscopic computerized tomography (microCT), showed the correlation between alveolar bone microstructural and skeletal BMD in pre- and postmenopausal women. In the study by Yamashita-Mikami et al., the premenopausal group had highly connective cancellous bone with thick interconnected trabeculae. In contrast, in the postmenopausal groups, there was cancellous bone with low connectivity and thin, dispersed and disconnected trabeculae [41]. Taken together, the use of PAN could play an important role in screening patients with osteoporosis, particularly postmenopausal women, because it is frequently supplemented as a part of routine oral diagnosis, and it is cheaper than DXA scan. When PAN is obtained, MCI, PMI, and MCW could be helpful for dentists to screen patients with undetected low BMD so that they can then be referred to medical professionals for bone densitometry [8, 10]. Our study confirms earlier publications that using MCI, the presence of a thinner or eroded mandibular cortex was more prevalent in postmenopausal women.

Determination of plasma OCN could be helpful to predict probable low BMD before performing bone densitometry. OCN levels could be a useful diagnostic tool to select patients with probable femoral neck or lumbar spine osteoporosis for BMD measurement [11, 17, 42, 43]. There were significant differences in OCN levels among osteoporosis, osteopenia, and healthy patients. The levels were most elevated in osteoporotic patients and had an inverse correlation with BMD [43]. Biver et al. performed a systematic analysis and reported that OCN levels between osteoporotic and nonosteoporotic control patients were different, and that this could be an argument to screen for radiographic vertebral fractures in asymptomatic patients with osteoporosis [44]. Moderate and negative correlations were found, mainly in postmenopausal women, between OCN level and BMD [44]. Liu et al. showed a positive correlation between estradiol and the BMD of the lumbar spine and the proximal femur, but a negative association between estradiol and OCN level [43]. In accordance with the present study, plasma OCN levels were significantly higher in postmenopausal versus premenopausal women. It is suggested that estrogen hormonal change in postmenopausal women can affect the function of bone metabolism, and as a result, can indirectly affect the OCN levels, which is consistent with our results. Singh et al. also reported the inverse correlation of OCN levels with femoral neck and lumbar spine BMD in postmenopausal women [42]. They indicated that serum OCN level is able to differentiate between postmenopausal women with normal BMD and postmenopausal women with osteopenia or osteoporosis. However, because the difference in OCN levels between osteopenic and osteoporotic women is not statistically significant, OCN levels alone cannot be used as a tool to differentiate between osteopenia and osteoporosis [42]. Recently, significant correlations were also shown between alveolar bone volume, trabecular bone number and spacing detected by microCT, and skeletal BMD with OCN [41]. Makker et al. observed a statistically significant association of mandibular BMD with OCN in postmenopausal women [11]. Serum OCN showed significantly higher levels in MCI C3 compared with MCI C1 [45], which is similar to our study. It can be hypothesized that general bone metabolism affects both alveolar bone and MCI, resulting in elevated OCN levels. Nevertheless, perhaps because of the small sample size, the current study did not show the association between OCN level and MCI after adjustment for age. Only postmenopausal status was related to elevated OCN levels. In the current study, we controlled many factors that might affect BMD. Circadian, fasting, and lifestyle factors (tobacco smoking and alcohol intake) as well as systemic disease and medication characterized by an acceleration of bone turnover were excluded. Therefore, it could be assumed in the present study that elevated plasma OCN was associated with postmenopausal status and possibly with the low mandibular BMD detected by MCI. Further study should be performed to confirm the inconsistent results among the plasma OCN, MCI, and postmenopausal status.

Regarding plasma OCN and periodontal status, very little data have been published. The relationship between OCN and AL was obscure. As AL increased, higher plasma OCN levels were detected in our study (*p* < 0.001, data not shown). However, the significant correlation disappeared after adjustment for age. Özçaka et al. reported that plasma OCN may not provide distinguishing data between participants with clinically healthy gingiva and chronic periodontitis [46]. Another study confirmed the results that OCN levels from the gingival crevicular fluid differed from systemic levels. OCN level in the gingival crevicular fluid correlates with periodontal but not with osteoporosis status, and systemic OCN levels were not related to periodontal status [47]. Conflicting results have been reported; Yoshihara et al. disclosed that the number of remaining teeth and serum OCN were negatively associated with the percentage of sites with AL ≥6 mm [45]. The relationship between plasma and gingival crevicular fluid OCN and periodontal disease should be further researched.

Plasma OCN level showed negative associations with BMI and BMD [44, 48]. Hence, overweight or obesity in postmenopausal women who had elevated levels of OCN in the current study may be explained by sedentary lifestyle rather than menopause itself. This assumption is supported by the study of Sternfeld et al., which showed that change in menopausal status was not associated with weight gain or significantly associated with increases in waist circumference [49]. Low BMD has also been shown to be associated with surrogate markers of cardiovascular disease (CVD), such as atherosclerosis or vascular abnormalities, after age, sex, BMI, or other vascular risk factors were adjusted [50, 51]. Postmenopausal women with low BMD or osteoporosis showed a twofold increased risk of vascular abnormalities including carotid artery calcification, CVD, and coronary artery disease [51]. Holvik et al. performed a cohort study and showed that a higher plasma OCN concentration was associated with an increased risk of CVD in women aged ≥75 years, and this hazard was not mediated by the established metabolic risk factors for CVD or by aortic calcification [52]. Our study showed related results; postmenopausal women had increased risk factors for CVD, including higher plasma OCN levels, overweight or obesity ratios, dyslipidemia, and impaired fasting blood sugar, when compared with premenopausal women. Uyl et al. found that after menopause, following estrogen withdrawal, the production and secretion of the proinflammatory cytokines interleukin-6, interleukin-1, and TNF-α increased. Inflammation is considered to play an important role in the process of atherosclerosis and CVD in individuals who have risk factors [50]. Likewise, postmenopausal women with a low BMD had one- to twofold increased risk of dying from CVD events, independent of traditional CVD risk factors [50]. Therefore, postmenopausal women who have increased levels of plasma OCN or decreased BMD scores should be screened for CVD risks.

This study has some limitations. The cross-sectional nature of the study limits its ability to make causal relationships. Selection bias might have occurred because the study group was a select group who presented for a medical examination and a further dental examination. Generalization of our results to other populations should therefore be made with caution. Besides this, a small number of participants were recruited for analyses. The median age of both studied groups was different though we used multivariate analysis in final data analyzing to control this confounding factor. The standard osteoporosis diagnosis by DXA was not performed; therefore, correlation with mandibular BMD was unable to be confirmed. Despite some limitations, the results are promising. As a result of the clinical and radiographic findings, dentists could improve the early detection of postmenopausal patients who have an increased risk of low BMD and refer them for further examination.

## 5. Conclusions

Clinical parameters such as oral mucosal dryness, an elevated number of teeth with periapical radiolucency, a high number of missing teeth, and thinning of the mandibular cortex seen on PANs were prevalent in postmenopausal women. Elevated plasma OCN and reduced mandibular BMD may help to predict osteopenia or osteoporosis in postmenopausal dental patients. Establishment of a good oral hygiene protocol is also imperative.

## Figures and Tables

**Table 1:** Personal and general health profile of participants according to pre- or postmenopausal status.

Variables	Participants		*p*
	Premenopause (*n* = 50)	Postmenopause (*n* = 42)	
Age (years)	43 (38, 45)	55 (51, 62)	<0.001
Body mass index plus waist circumference (*n* = 66)			
Normal weight	21	4	0.005
Overweight or obesity	20	21	
Cholesterol (mg/dL) (*n* = 92)			
<200	33	13	0.002
≥200	17	29	
High-density lipoprotein cholesterol (mg/dL) (*n* = 83)			
≥50	26	17	0.275
<50	19	21	
Low-density lipoprotein cholesterol (mg/dL) (*n* = 92)			
<100	14	4	0.035
≥100	36	38	
Triglyceride (mg/dL) (*n* = 83)			
<150	29	18	0.128
≥150	16	20	
Fasting plasma glucose (mg/dL) (*n* = 83)			
<100	35	21	0.036
≥100	10	17	
Systolic blood pressure (mmHg) (*n* = 82)			
<130	33	23	0.343
≥130	12	14	
Diastolic blood pressure (mmHg) (*n* = 82)			
<85	34	30	0.601
≥85	11	7	
Glomerular filtration rate (mL/min/1.73 m^2^) (*n* = 82)			
≥90	43	27	0.221
<90	5	7	
Plasma osteocalcin levels (ng/mL)	234.77 (171.57, 303.36)	425.62 (312.88, 508.19)	<0.001
Alcohol consumption			
Never/former	36	27	0.642
Current	4	1	
Smoking			
Never/former	42	29	1.000
Current	1	0	

**Table 2:** Oral disease of participants according to postmenopausal status.

Variables	Participants		*p*
	Premenopause	Postmenopause	
Average number of missing teeth	1 (2, 4)	6 (2, 16)	0.008
Mean attachment loss (mm)	2.1 (1.7, 2.4)	2.6 (2.2, 3.7)	<0.001
Alveolar bone loss (*n* = 89)			
<25%	46	31	0.031
≥25%	3	9	
Number of periapical radiolucencies (*n* = 75)			
Absence	36	21	0.006
1–4	7	11	
Clinical score of oral dryness (*n* = 92)			
0	25	8	0.005
1–3	24	30	
≥4	1	4	

**Table 3:** Mandibular bone density of participants from various indices according to postmenopausal status.

Variables	Participants		*p*
	Premenopause (*n* = 46)	Postmenopause (*n* = 35)	
Right mandibular cortical index			
1	45	22	<0.001
2	1	12	
3	0	1	
Left mandibular cortical index			
1	45	22	<0.001
2	1	12	
3	0	1	
Right panoramic mandibular index	0.28 ± 0.06	0.26 ± 0.07	0.405
Left panoramic mandibular index	0.27 ± 0.06	0.27 ± 0.08	0.619
Right mandibular cortical width (mm)	3.50 ± 0.98	3.39 ± 0.81	0.630
Left mandibular cortical width (mm)	3.61 ± 0.64	3.40 ± 0.93	0.250
Right fractal dimension	1.18 ± 0.11	1.19 ± 0.10	0.457
Left fractal dimension	1.23 ± 0.11	1.18 ± 0.97	0.033

**Table 4:** Association of osteocalcin levels with participants' general and oral health status.

Osteocalcin levels										
Variables	Model 1		Model 2		Model 3		Model 4		Model 5	
	*β*	*p*	*β*	*p*	*β*	*p*	*β*	*p*	*β*	*p*
Age	0.636	<0.001	0.289	0.043	0.302	0.031	0.272	0.064	0.264	0.073
Presence of postmenopausal status			0.461	0.002	0.500	0.001	0.487	0.001	0.504	0.001
Presence of overweight or obesity					−0.171	0.073	−0.169	0.079	−0.165	0.086
Mandibular cortical index							0.072	0.501	0.096	0.383
Mean alveolar bone loss									−0.097	0.310

*β* values were derived from a multiple linear regression analysis adjusted for age, postmenopausal status, presence of overweight or obesity, mandibular cortical index, and mean alveolar bone loss.

## Data Availability

The data that support the findings of this study are available on request from the corresponding author. The data are not publicly available because they contain information that could compromise research participant privacy.
